# Anti-calmodulins and Tricyclic Adjuvants in Pain Therapy Block the TRPV1 Channel

**DOI:** 10.1371/journal.pone.0000545

**Published:** 2007-06-20

**Authors:** Zoltán Oláh, Katalin Jósvay, László Pecze, Tamás Letoha, Norbert Babai, Dénes Budai, Ferenc Ötvös, Sándor Szalma, Csaba Vizler

**Affiliations:** 1 Institute of Biochemistry, Biological Research Center of the Hungarian Academy of Sciences, Szeged, Hungary; 2 Acheuron Hungary Ltd., Szeged, Hungary; 3 Acheuron Pharmaceuticals Inc., San Diego, California, United States of America; 4 Department of Medical Chemistry, Faculty of General Medicine, University of Szeged, Szeged, Hungary; 5 Department of Experimental Zoology and Neurobiology, University of Pécs, Pécs, Hungary; 6 Department of Biology, Gyula Juh‡sz Faculty of Education, University of Szeged, Szeged, Hungary; The Rockefeller University, United States of America

## Abstract

Ca^2+^-loaded calmodulin normally inhibits multiple Ca^2+^-channels upon dangerous elevation of intracellular Ca^2+^ and protects cells from Ca^2+^-cytotoxicity, so blocking of calmodulin should theoretically lead to uncontrolled elevation of intracellular Ca^2+^. Paradoxically, classical anti-psychotic, anti-calmodulin drugs were noted here to inhibit Ca^2+^-uptake *via* the vanilloid inducible Ca^2+^-channel/inflamatory pain receptor 1 (TRPV1), which suggests that calmodulin inhibitors may block pore formation and Ca^2+^ entry. Functional assays on TRPV1 expressing cells support direct, dose-dependent inhibition of vanilloid-induced ^45^Ca^2+^-uptake at µM concentrations: calmidazolium (broad range)≥trifluoperazine (narrow range)>chlorpromazine/amitriptyline>fluphenazine>>W-7 and W-13 (only partially). Most likely a short acidic domain at the pore loop of the channel orifice functions as binding site either for Ca^2+^ or anti-calmodulin drugs. Camstatin, a selective peptide blocker of calmodulin, inhibits vanilloid-induced Ca^2+^-uptake in intact TRPV1^+^ cells, and suggests an extracellular site of inhibition. TRPV1^+^, inflammatory pain-conferring nociceptive neurons from sensory ganglia, were blocked by various anti-psychotic and anti-calmodulin drugs. Among them, calmidazolium, the most effective calmodulin agonist, blocked Ca^2+^-entry by a non-competitive kinetics, affecting the TRPV1 at a different site than the vanilloid binding pocket. Data suggest that various calmodulin antagonists dock to an extracellular site, not found in other Ca^2+^-channels. Calmodulin antagonist-evoked inhibition of TRPV1 and NMDA receptors/Ca^2+^-channels was validated by microiontophoresis of calmidazolium to laminectomised rat monitored with extracellular single unit recordings *in vivo*. These unexpected findings may explain empirically noted efficacy of clinical pain adjuvant therapy that justify efforts to develop hits into painkillers, selective to sensory Ca^2+^-channels but not affecting motoneurons.

## Introduction

Recently, we and several other groups noted that TRPV1, the vanilloid ligand gated member of the TRP (transient receptor potential) super family, localizes both in the plasma membrane (TRPV1_PM_) and endoplasmatic reticulum (TRPV1_ER_) membranes [1]–[4], and upon ligand binding they release Ca^2+^ to the cytosol. These pools are inducible with: i) exo-, or endovanilloid ligands [5], [6], ii) proton [7], [8], iii) phosphorylation *via* intracellular signaling by protein kinases [9]–[11] and iv) heat (42–49°C) [12], [13]. Dynamics of TRP cation channel opening and closing has remained largely unknown, although various mechanisms have been proposed [11], [14]. Due to lack of purification and crystallization protocol the three-dimensional (3D) structure information is scarce, the initial conformation change and subsequent steps leading to pore opening/closing has yet to be elucidated. Better understanding of transmembrane proteins is hampered by process development such as high scale production, solubilization, and purification, which preserves the native state and function. As in case of many transmembrane domain proteins/ion channels, 3D structure of TRPV1 is still subject of intense research [15], [16].

Calmodulin is the most well-known calcium binding protein, which is ubiquitous and preserved in the eukaryotic cell, either human, animal, fungal or plant. Ca^2+^, among others, selectively interacts with the so-called “EF-hand”/Ca^2+^-binding protein motifs located either intracellularly or extracellularly [17]. Dose-dependent interaction of Ca^2+^ with calmodulin elicits a robust conformational change that exposes hidden hydrophobic domains required for subsequent effects on down-stream protein targets [18], [19]. Ca^2+^- calmodulin complex, formed upon entry of Ca^2+^ to the cytosol resulting in elevation of intracellular free calcium [Ca^2+^]_i_ can turn on/off different enzymes and ion channels. Camstatin, a recently found conserved polypeptide motif in PEP-19, neurogranin and neuromodulin, has been noted to enhance dissociation of Ca^2+^ from calmodulin [20] and disable interaction with down-stream targets [21]. These observations suggest that one of the major functions of calmodulin would be to buffer and/or neutralize the rapid increase of [Ca^2+^]_i_, thus to prevent excitotoxicity. Serving as a shut off valve on broad-spectra of Ca^2+^-channels, Ca^2+^- calmodulin protects Ca^2+^-overload-induced cell death, either due to necrotic or apoptotic mechanisms. The specific intracellular sites has been identified but the exact mechanism of calmodulin binding is still debated [22], [23].

The Ca^2+^- calmodulin mediated feedback due to increased [Ca^2+^]_i_, has recently been elucidated in detail in case of the TRP3 channel. It has been noted that upon Ca^2+^-depletion, IP_3_R, a sensor of Ca^2+^-load of ER, directly interacts and props the TRP3_PM_ channel open by the so-called “store operated Ca^2+^-entry” mechanism. Indeed, one or two specific domains of IP_3_R can interact with cognate sites of TRP3_PM_ and contribute to opening of the pore. However, both Ca^2+^- calmodulin and cytoplasmic domain of IP_3_R_ER_ competes for an overlapping site and either open or close the given TRP channel, respectively and the preference only depends on the levels of [Ca^2+^]_i_. In fact, calmodulin, upon saturation with Ca^2+^ displaces IP_3_R_ER_, which leads to termination of store operated Ca^2+^ entry. However, Ca^2+^- calmodulin can be displaced by excess synthetic peptides, derived either from the competitive IP_3_R motif or from the heterologous myosin light chain kinase. The former is known to block IP_3_R_ER_ binding to TRP3_PM_ by direct competition, whereas, cognate domain from myosin light chain kinase, as well as calmidazolium, inhibit the interaction indirectly, due to prevention of Ca^2+^ loading of calmodulin. It is conceivable that either mechanism can serve as a shut off valve of TRP3_PM_. In general, either disruption of a TRP-Ca^2+^-channel interaction or block of Ca^2+^-feedback by anti- calmodulin agents can deregulate store operated Ca^2+^ entry and cause eventually excitotoxicity and cell death by Ca^2+^-overload [24], [25]. Indeed, application of calmidazolium to HL-60 cells has recently been shown to increase [Ca^2+^]_i_, which is consistent with disrupted Ca^2+^- calmodulin feedback regulation [26]. Ca^2+^- calmodulin-mediated termination of Ca^2+^-entry is not confined to TRP channels only [27], rise of [Ca^2+^]_i_ also shuts off M-, and L-type voltage-gated channels. Opening of Ca^2+^-activated, small conductance K^+^ (SK) channels is elicited by calmodulin binding to the C-terminus. Further elevation of [Ca^2+^]_i_ and saturation of calmodulin with Ca^2+^ inactivate the pore opening mechanism [28], [29].

Consistent with this Ca^2+^-feedback theory, anti- calmodulin agents, such as calmidazolium are expected to suspend the feedback on Ca^2+^-entry, and thus potentiate elevation of [Ca^2+^]_i_ to toxic levels [30]. Classical antipsychotic drugs, such as trifluoperazine, chlorpromazine and fluphenazine also target calmodulin, so they were also hypothesised to deregulate agonist-induced Ca^2+^ increase. To test this hypothesis and better understand channel function of TRPV1, we carried out experiments with tricyclic anti-psychotic calmodulin antagonist drugs and other selective inhibitors of calmodulin. Our new data, in lieu of expectations, suggest that *bona fide* calmodulin antagonists blocks and do not promote Ca^2+^-transport *via* TRPV1.

To address paradoxical effects of anti- calmodulin agents, we hypothesized that these compounds might not enter the cell, but inhibit the pore opening of pain/TRPV1 channel, most likely directly at the cation filter site, located at the extra-cellular orifice of the Ca^2+^-channel. In fact, sequence comparisons of various TRP channels suggest that a short acidic amino acid stretch at the pore loop of TRPV1 resembles to “EF-hand” and “*Excalibur*” motifs, both identified previously in calmodulin and calmodulin-like proteins, respectively [31]. Since direct sequence homology was not that obvious, structure and function of TRPV1 was probed with various anti-calmodulin agents such as antipsychotic drugs and homologues with basic pharmacophore similar to that identified in calmidazolium. Functional assays were carried out in TRPV1-NIH3T3 cells permanently expressing the receptor ectopically, and in primary cultures from embryonic rat dorsal root ganglia (DRG), enriched in TRPV1^+^ nociceptor/pain neurons.

Consistent with our hypothesis, but contrary to the Ca^2+^-calmodulin feedback theory, a set of anti-calmodulin agents acutely inhibited the vanilloid/capsaicin–induced Ca^2+^-uptake in TRPV1 expressing cells and neurons. Extracellularly added camstatin peptide, an antagonist of calmodulin, also blocked capsaicin–induced Ca^2+^-uptake in intact cells. Studies carried out at the cellular levels and in animal pain models were in concert with previously noted analgesic actions of calmodulin-antagonists determined *in vivo* [32], but we gave here new evidence that calmodulin antagonists can directly block ion channel function of TRPV1. We suggest that the analgesic effect of these calmodulin antagonists is independent of conventional intracellular targets and we propose a specific site of action located extracellularly [23]. Pharmacophores of various calmodulin inhibitors studied here may yield discovery of novel analgesics in the near future [33].

## Results

**Figure 1:**
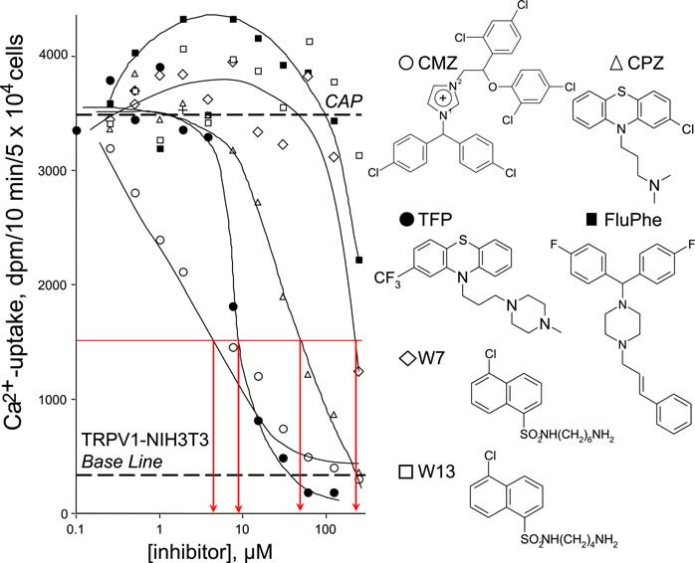
Antagonist activity of Ca^2+^-calmodulin inhibitors in TRPV1-NIH3T3 cells. ^45^Ca^2+^-uptake experiments were carried out in 96 well plates with robotic liquid handling. Cells were co-incubated with capsaicin (capsaicin, ED_200_ = 2 µM) in the presence of different concentrations of Ca^2+^- calmodulin inhibitors. Calmidazolium (CMZ)>trifluoperazine (TFP)>chlorpromazine (CPZ) were identified as full antagonists of capsaicin-induced Ca^2+^-uptake, in the micromolar range, while fluphenazine (FluPhe), W7 and W13 were determined as partial or weak inhibitors. Similar efficacy order was determined in two additional experiments, carried out in duplicate samples.

**Figure 2:**
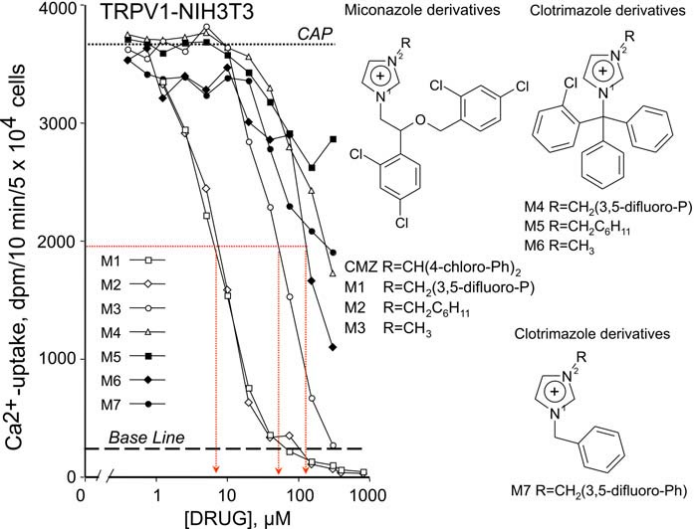
Screening of other calmidazolium analogs (M-set). Among miconazoles, clotrimazole, and N-benzylimidazole compounds (M1–M7) calmidazolium was the most potent inhibitor in TRPV1-NIH3T3 cells. Each point on the graph is the average of triplicate determinations. Chemical structure of miconazoles, clotrimazole, and N-benzylimidazole compounds used in these studies are indicated. Experiments were repeated two additional times in triplicate with similar results.

To better understand potential effect of Ca^2+^- calmodulin inhibitors on nociception, activity of various phenothiazines were studied at the molecular levels in TRPV1-NIH 3T3 cells, in which the pain signal was mimicked with capsaicin-induced ^45^Ca^2+^-uptake (Fig. 1). Assays were carried out in 2×10^−5^ M extracellular Ca^2+^ in 96 well plate formats at room temperature (22°C) for 10 min with robotic liquid handling. Briefly, cells were co-incubated with capsaicin for vanilloid-induced opening of TRPV1. Various Ca^2+^- calmodulin inhibitors were co-incubated in progressively increasing concentration in the 2 µM capsaicin supplemented incubation medium. Increased Ca^2+^-uptake was expected, due to a severe damage of shut off valve mechanism caused by the presence of calmodulin antagonists. Contrary to this theory, detailed referring to other TRP channels in the introduction, we noted that the tested calmodulin inhibitors inhibited the capsaicin-induced Ca^2+^-uptake in TRPV1-NIH3T3 cells with varying potency: calmidazolium (IC_50_ = 7 µM)>trifluoperazine (IC_50_ = 9 µM)>chlorpromazine (IC_50_ = 70 µM)>W-7 (IC_50_∼200 µM)>fluphenazine (IC_50_∼250 µM), and W-13 (IC_50_>300 µM), and [Ca^2+^]_i_ did not accumulate in these cells at even higher concentrations of calmodulin inhibitors. The same agents were also tested in the absence of capsaicin, where none of them induced Ca^2+^ influx, i.e., they did not activate the vanilloid receptor. For the sake of clarity these curves were omitted from Fig 1. and the following figures also (Fig 2–6). Efficacy of calmidazolium in the µM scale was also validated in a human TRPV1-HaCaT keratinocyte line (data not shown). Interestingly, fluphenazine, but less pronouncedly both W13 and W7 increased Ca^2+^-uptake in the presence of 2 mM capsaicin at low concentrations. Similar cooperative effect was noted previously between [^3^H] resiniferatoxin and different antipsychotic and antidepressant drugs [34]. We hypothesized that calmodulin antagonists and some registered drugs may exert their blocking effect with another mechanisms on TRPV1 and not necessarily on their conventional target. Acting extracellularly, the inhibitors prevent TRPV1 activation, therefore the intracellular calmodulin might be secondary and has little if any effect on [Ca^2+^]_i_ signal induced by inflammatory pain agonists either exo-, or endogenous.

To better define the quantitative structure-activity relationship (qSAR), other miconazole compounds, analogs of the commercially available calmidazolium, were functionally assayed in cells expressing TRPV1. A small dedicated library of miconazoles, clotrimazole and N-benzylimidazole compounds, referred to as the M-set (see M1–M7 in Fig. 2) were tried. All of these analogues of calmidazolium originally were synthesized to develop inhibitors of L-type Ca^2+^-channels [26]. Dose-response analysis of capsaicin-induced Ca^2+^-transport carried out in TRPV1-NIH 3T3 cells eliminated N-benzylimidazole and clotrimazole compounds form further studies, since both groups showed either only partial inhibition or less favorable IC_50_ than calmidazolium and other miconazoles (i.e. M1/M2 = 8 µM>M3∼75 µM) homologues (Fig. 2).

**Figure 3 pone-0000545-g003:**
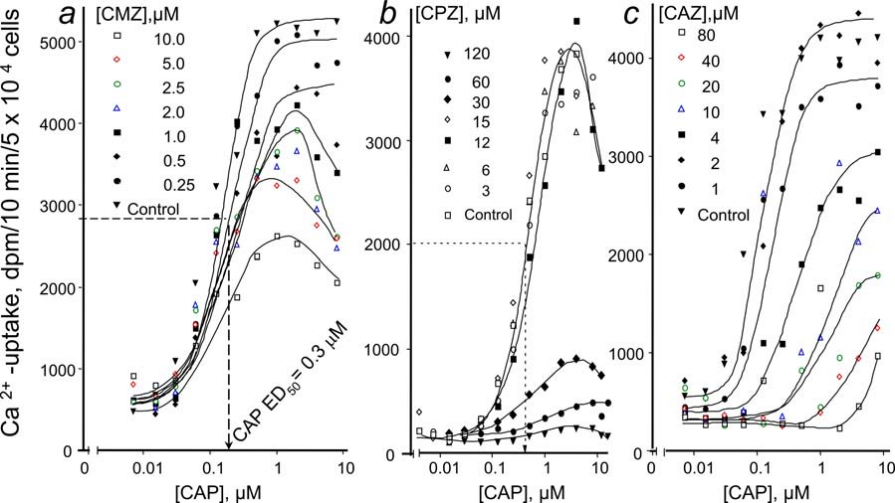
Kinetics of Ca^2+^-transport inhibition by calmidazolium (CMZ), chlorpromazine (CPZ) and capsazepine (CAZ) in TRPV1-NIH3T3 cells, induced by increasing concentrations of capsaicin. Increasing doses of calmidazolium decreased V_max_ of Ca^2+^-transport, however, affinity of TRPV1 to capsaicin remained constant, as indicated. Likewise, chlorpromazine showed distinctive, non-competitive inhibition kinetics, similar to that determined to calmidazolium, consistent with a channel blocking mechanism on TRPV1. In contrast to calmidazolium and chlorpromazine, capsazepine, a *bona fide* vanilloid antagonist shifted the capsaicin dose-response curves right, however, above 1 µM behaved as a mixed kinetics inhibitor, also decreased V_max_. Experiments were repeated two additional times in duplicates with similar results.

Further kinetic analysis of calmidazolium addressed the potential mechanism of inhibition in TRPV1 expressing cells. A dramatic decrease in the maximal velocity (V_max_) of capsaicin-induced Ca^2+^-uptake, but not the ED_50_ of capsaicin (0.3 mM of capsaicin) was determined. With progressively increasing concentrations of calmidazolium the V_max_ gradually decreased, which also showed a characteristic maxima instead of a plateau due to a potential interaction with capsaicin on TRPV1. Above a calmidazolium concentration of = 2.5 µM, Ca^2+^-transport was almost completely abolished (Fig. 3a). Distinctive drop in V_max_ were prominent at low concentrations of calmidazolium, which is rather consistent with a channel blocking mechanism, than competition for the capsaicin-binding site. Likewise calmidazolium, chlorpromazine showed distinctive, non-competitive inhibition kinetics of V_max_ (Fig. 3b).

Capsazepine, a known competitive inhibitor at the intracellular vanilloid binding site, typically shifts the dose-response curve right (Fig. 3c), but at low concentrations does not affect V_max_, similar to that determined previously by a non-penetrating ligand of TRPV1 [35]. Inhibition kinetics of calmidazolium and chlorpromazine were markedly differed from that determined to capsazepine, a *bona-fide* vanilloid mimetic antagonist. A decline in V_max_, relative to a plateau might be a sign of allosteric interaction of calmidazolium and chlorpromazine with other domain(s) (Fig. 3a and 3b) that may affect access of capsaicin to TRPV1.

**Figure 4 pone-0000545-g004:**
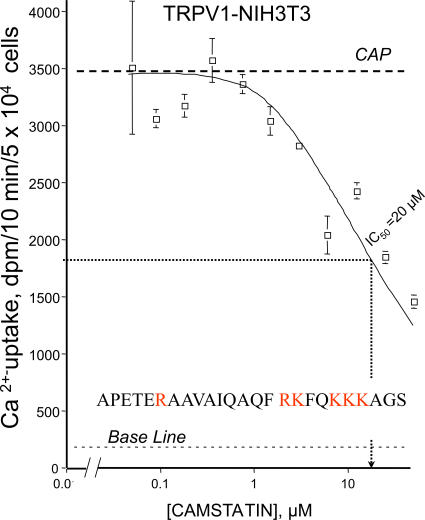
Effect of a non-membrane permeable inhibitor of calmodulin on TRPV1-mediated Ca^2+^-transport. Camstatin, a recently identified, selective, 25-mer polypeptide blocker of calmodulin, was employed, which inhibited capsaicin-induced Ca^2+^-uptake with comparable activity (IC_50_ = 20 µM) determined previously to other, more conventional antagonists, of calmodulin in TRPV1-NIH3T3 cells. Results with camstatin suggests calmodulin-like structure at the extracellular domains of TRPV1. Experiments were repeated two additional times in triplicate with similar results.

From these experiments we hypothesized that maybe a calmodulin-like motif of TRPV1 is recognized extracellularly, therefore, a calmodulin antagonist with poor membrane permeability can exert inhibition without entering the cell. To address extracellular targeting of TRPV1, camstatin, a recently identified, selective anti- calmodulin peptide was coincubated with capsaicin in the Ca^2+^-uptake medium. Likewise conventional calmodulin inhibitors, camstatin inhibited capsaicin -induced Ca^2+^-transport (IC_50_ = 11 µM) within 10 min of the assay in TRPV1-NIH3T3 cells. Prompt inhibition of inducible Ca^2+^-uptake suggested a rapid interaction with a potential extracellular docking site(s) of TRPV1 (Fig. 4). As a control, CAMKII kinase inhibitor peptide, similar in size to camstatin but distinct in biological activity, did not block capsaicin-induced Ca^2+^-uptake (data not shown). Experiments with peptides suggested that camstatin and other anti-calmodulin agents indeed recognize an extracellular domain of TRPV1.

**Figure 5 pone-0000545-g005:**
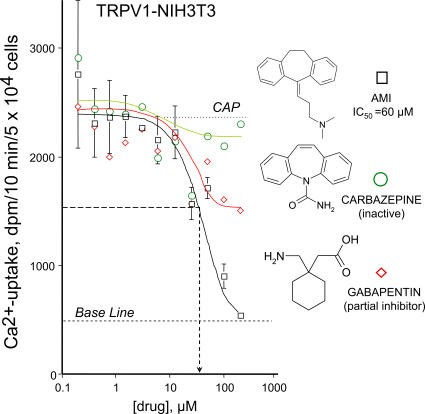
Activity of other drugs, similar to chlorpromazine and trifluoperazine, on TRPV1. Amitriptyline (AMI), carbamazepine and gabapentine were tried in cell based capsaicin-induced Ca^2+^-uptake experiments. Among these substances amitriptyline, a tricyclic analogue of phenothiazines, was the best inhibitor, however, carbamazepine with shorter side chain than amitriptyline was determined inactive. Gabapentine showed only partial inhibition on TRPV1. Similar results were obtained in two additional experiments carried out in triplicate.

To make initial structure activity relationship studies of TRPV1 inhibitors more complete and check efficacy of a clinically tried drugs, amitriptyline, a known antidepressant in human [36], gabapentine, a recently commercialized painkiller drug, and carbamazepine, an amitriptyline analog prescribed for patients with chronic back pain were tried in capsaicin-induced Ca^2+^-uptake experiments. Among these substances amitriptyline, a structure analogue of phenothiazines, was determined the best inhibitor (IC_50_∼60 µM), however, its effect was significantly less prominent than that of trifluoperazine (IC_50_<10 µM). Carbamazepine, with shorter side chain than that in amitriptyline was completely inactive in this assay, while gabapentine showed partial inhibition (Fig. 5). SAR of these drugs highlights the role of the aliphatic extension tethering the tricyclic ring of these types of analogs.

**Figure 6:**
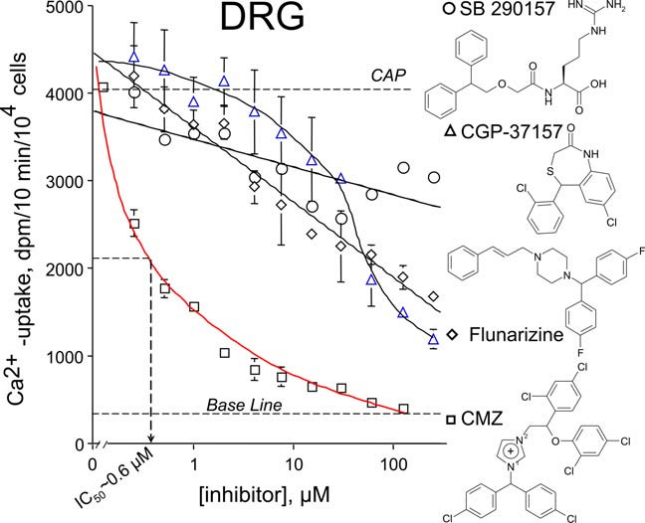
Efficacy of Ca^2+^- calmodulin inhibitors in rat primary DRG cultures. Neuron cultures derived from embryonic rat DRGs readily show inducible (10 fold over base line) activation with vanilloids due to endogenous expression of TRPV1. As with the TRPV1-NIH 3T3 cells, calmidazolium was determined the most effective inhibitor of capsaicin-induced Ca^2+^-uptake. As controls of specificity, flunarizine, SB 290157, and CGP 37157 showed only partial blocking activity in DRG neurons. Similar results were obtained in two additional experiments carried out in triplicate.

To test selectivity and efficacy in an *in vivo* target, calmidazolium, the best channel blocker of capsaicin-induced Ca^2+^-uptake in TRPV1-NIH3T3 cell was tried in sensory neurons, expressing TRPV1 endogenously. Primary cultures were prepared from embryonic (E14) rat DRGs. Along with calmidazolium other polycyclic compounds were included in the vanilloid-induced ^45^Ca^2+^-transport assays. SB 290157, a selective, high affinity, competitive antagonist of the anaphylatoxin C3a receptor, CGP-37157, a cell-permeable benzodiazepine inhibitor of mitochondrial Na^+^/Ca^2+^ exchanger, and flunarizine, a piperazine derivative blocker of T-type cardiac Ca^2+^ channels were included in assays in DRG cultures. All of them were far less potent than calmidazolium. Moreover, calmidazolium inhibited capsaicin-induced Ca^2+^-uptake in sensory neuron cultures with a higher potency than that was determined in TRPV1-NIH3T3 cells (i.e. 0.6 µM vs. ∼2 µM, respectively) (Fig. 1 and Fig. 6). Although CGP-37157 and flunarizine were determined partially active, in a relative broad concentration range, calmidazolium was determined far more potent (∼3 fold) inhibitor in DRG cultures enriched in nociceptive neurons with TRPV1 immunoreactivity (data not shown) (Fig. 6).

**Figure 7:**
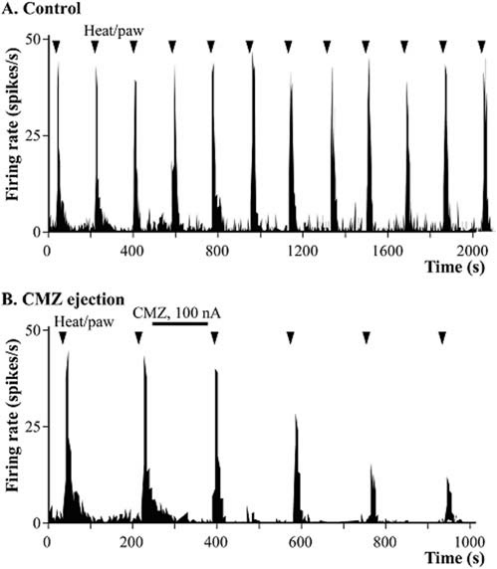
Heat-induced pain signal inhibition of the best Ca^2+^- calmodulin inhibitor in the rat. Ratemeter recordings showing the inhibitory effects of calmidazolium (CMZ) on responses of a spinal dorsal horn neuron to noxious heat delivered to its cutaneous receptive field on the paw. Panel A: Heat-evoked responses evoked in every 3 min during the control period.. Panel B: Effects of iontophoresed CMZ on the heat-evoked responses. Similar results were obtained in five additional experiments. A representative recording is shown.

To validate *in vivo* efficacy of calmidazolium, experiments were carried out in a total of 22 nociceptive specific (NS) or wide dynamic range (WDR) spinal dorsal horn neurons in 16 lightly anesthetized rats. Effects of calmidazolium were studied on the heat evoked responses of dorsal horn neurons using noxious peripheral heat stimuli delivered in every 3 min in the cutaneous receptive field of the hind paw. After establishing stable baseline control responses, calmidazolium was iontophoresed (i.e. with 100 nA) from one of the barrels of the compound electrode. Heat-evoked responses of dorsal horn neurons were significantly reduced the responses to peripheral noxious heat to a mean of 34±23% (p<0.01, n = 6) of pre- calmidazolium baseline control (Fig. 7). The calmidazolium evoked inhibition reached its maximum within 10 to 15 min after ejection and lasted throughout the entire recording periods (about 2 hrs) of the experiment. The monitored cells had excitatory receptive fields located on one of the hind paws. These cells usually had little spontaneous activity, located between 100–600 µm from the surface of the dorsal horn, as estimated by microdrive readings. Pain signaling, evoked by noxious heat to cutaneous receptive field of the paw were most likely transduced by TRPV1 expressing nociceptive neurons but occurrence of other heat sensors, such as TRPV2, the super heat receptor, could not be rule out. Likewise to that determined previously in TRPV1-NIH3T3 cells, TRPV1-HaCaT keratinocyte lines and primary culture of nociceptive neurons, calmidazolium inhibited the ratemeter recorded signals of radiation heat pain in the rat (Fig. 7). Iontophoretically ejected calmidazolium to the site of recordings validated potent inhibitor activity of a calmodulin antagonist *in vivo*, in the spinal dorsal horn neurons that noted previously in TRPV1 expressing cells.

**Figure 8:**
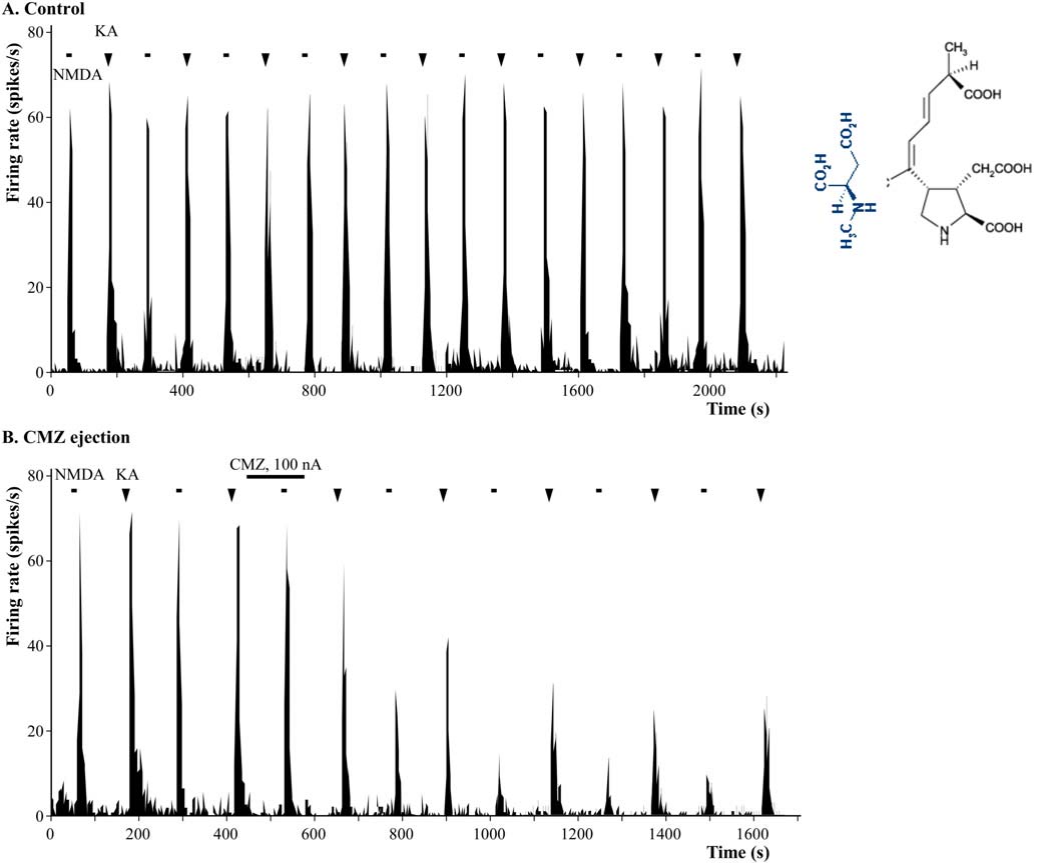
Calmidazolium (CMZ) differentially bocks NMDA-, and kainic acid (KA)-induced pain-responses. NMDA and kainic acid were iontophoresed, sequentially, 2 min apart using −85 nA and −30 nA, respectively, then calmidazolium was ejected as shown, which markedly inhibited the initial pain signals at the levels of dorsal horn neurons. Note the differential effects of calmidazolium on NMDA responses versus kainic acid responses. Panel A: Responses to NMDA and kainic acid iontophoresed alternately in every 2 min. Panel B: Effects of iontophoresed calmidazolium on the responses to these excitatory amino acids during the control period. Panel B: Differential effects of calmidazolium on the NMDA- versus the kainic acid-evoked responses. Similar results were obtained in fifteen additional experiments. A representative recording is shown.

**Figure 9:**
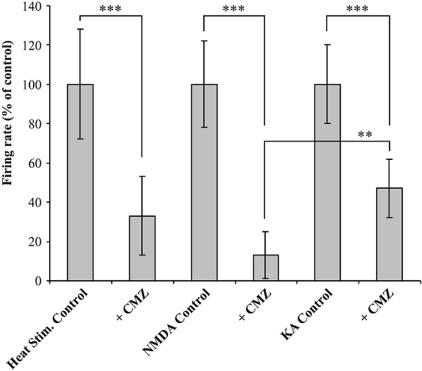
Summary of the effects of calmidazolium (CMZ) on the responses to heat or to iontophoresed excitatory amino acids of spinal dorsal horn neurons of nociceptive specific (NS) or wide dynamic range (WDR) types. Note the decrease of the responses in the presence of calmidazolium. Asterisks denote significant differences by ANOVA (**p<0.05, ***p<0.01) as compared to their respective controls or the significant difference between the decrease of NMDA- and kainic acid -evoked responses in the presence of calmidazolium. See details in the text.

In another set of experiments, effects of calmidazolium were investigated on the responses of dorsal horn neurons evoked by NMDA and kainic acid agonists of different glutamate receptors, well characterized transducers of pain signals, more centrally. Neuronal firing responses to iontophoresed NMDA or kainic acid were typically fast in onset and short in duration (Fig. 8). Ejection currents for these excitatory agents were delivered alternately in every 2 min and were selected to produce a maximal peak height of 50–70 spikes/s which corresponded to 300–400 action potentials per stimulation epoch. Iontophoretic application of calmidazolium produced significant reduction in responses to both NMDA and kainic acid. Maximal inhibition of NMDA- or kainic acid -evoked responses were reached 15–20 min after calmidazolium application and remained so during the rest of the 2 hour long experiment. Responses to NMDA were more pronounced, reduced the initial pain signals to 13±12% (p<0.01, n = 16), however, responses to kainic acid were moderate, inhibited the pre- calmidazolium baseline control responses to 47±20% (p<0.01, n = 16). There was a significant difference between the decreases of responses to NMDA versus kainic acid in the presence of calmidazolium (13±12% versus 47±20%, respectively, p<0.05) (Fig. 9).

## Discussion

Of important finding of these studies is that classical phenothiazines/antipsychotic drugs, all of them calmodulin inhibitors, also can serve as blocker of current *via* the Na^+^/Ca^2+^-channel of TRPV1, the specific transducer of heat/vanilloid-induced inflammatory pain. Among these drugs amitriptyline (i.e. Elavil®/Teperin®) has clinically been tried in adjuvant therapy, and noted effective in post-herpetic neuralgia and painful diabetic neuropathy [37]–[39]. Although previously was validated in clinical pain, amitriptyline (IC_50_ = 60 µM) not ranked among the most potent inhibitors of TRPV1 channel in our cell-based assay.

**Figure 10:**
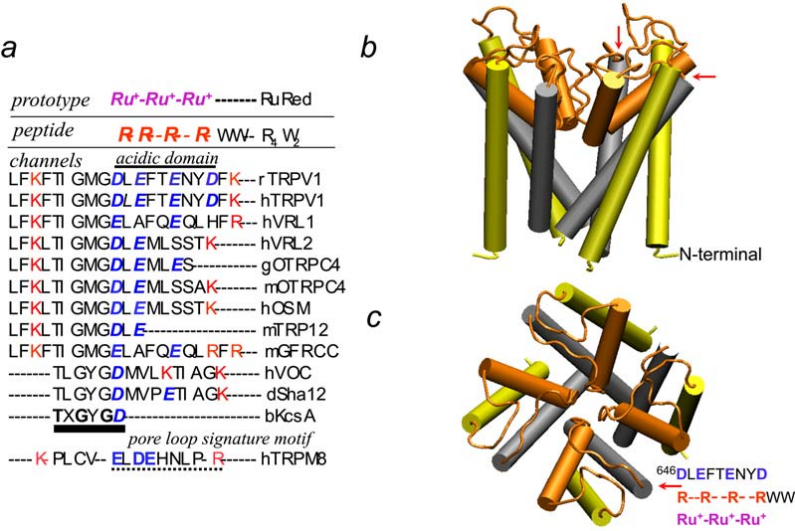
(*a*) Homologous portions of various TRP channels, near the border of the pore loop and the 6^th^ transmembrane domain were aligned with a validated R_4_W_2_ peptide similar in biochemical character to ruthenium red. An acidic tetrad motif *D*X*E*XX*E*XX*D* which can bind the positively charged peptides in human and rat TRPV1, is absent in TRPV2/VRL1 and TRPV3, (both close homologues of TRPV1) as well as in distantly related TRPs and bKcsA, a bacterial cation channel. An acidic sequence, partially similar to the heat sensitive TRPs, is present in the cold responsive TRPM8/CMR1. Distant TRPV homologues do not share the acidic tetrad motif either, such as g/mOTRPC4 and hOSM, nonselective cation channel orthologues from *Gallus gallus* (chicken), mouse, and human, respectively that confer sensitivity to extracellular osmolarity, mTRP12, another osmotically activated TRP channel from mouse; mGFRCC, mouse growth factor receptor coupled channel; hVOC, *Homo sapiens* Kv4.3 potassium channel; dSha12, a “shaker-like” potassium channel from *Drosophila melanogaster*. (*b*) The TM5-pore loop-TM6 region of TRPV1 is analogous to the “inverted teepee”, *pore*-forming domain of bKcsA. Side-view of the TRPV1 tetramer channel depicts the hypothetical pore at the middle. Arrows point to the putative ruthenium red/R_4_W_2_ binding site in each TRPV1 subunits of the tetramer. (*c*) To better represent the simulated quaternary structure of TRPV1, a view perpendicular to the plasma membrane is generated with the homo-tetrameric TRPV1 domain fragments. The position of acidic domain is noted by an arrow in a single subunit in this view of the model.

**Figure 11:**
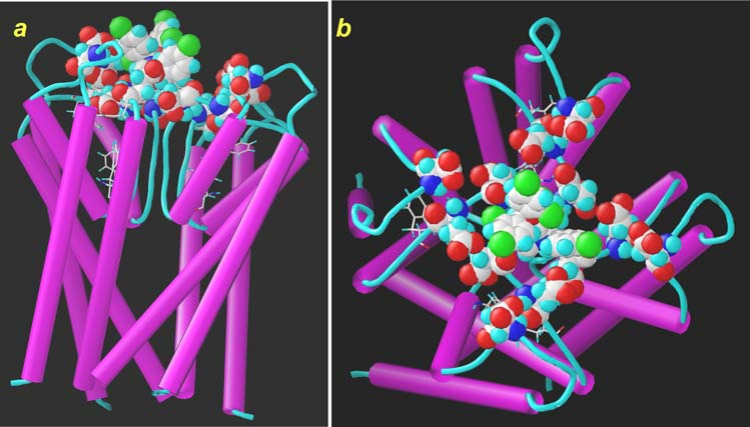
A docking of CMZ to pore loop domain of TRPV1. Symmetrical arrangement of the 5 and 6 TM helices (magenta) in the tetrameric TRPV1 receptor complex is shown. Aromatic residues within the 20 Å proximity to the inhibitor are shown by sticks, and the Asp and Glu residues as well as the ligand by spacefilled models. Color codes for the atoms: grey, C; cyan, H; blue, N; red, O; green, Cl. The pore loop, localized in between transmembrane domain 5 and 6 of TRPV1 is shown with the specific docking site of the positively charged CMZ. (*a*) side-view and (*b*) a view perpendicular to the cell membrane were generated after docking of CMZ. Negatively charged “acidic domain” of TRPV1 in the homotetramer may serve as ideal nest for channel blockers such as CMZ>trifluoperazine>chlorpromazine/amitriptyline, as well as, ruthenium red and R_4_W_2_, all charged oppositely. The rule is that more basic is an anti- calmodulin substance that more attracted to the acidic moieties of the “nest” by electrostatic forces near to the entrance (i.e. ion filter) of the pore. It is a tendency that longer is the hydrophobic side chain tethered to the tricyclic core better is the fit inside the pore, such as determined with amitriptyline and carbamazepine.

Migraine might be treated with rationally chosen calmodulin antagonist drugs currently available. Novel drug candidates such as calmidazolium analogs (M-set) may even more promising in this respect. Even when a patient is not clinically depressed, antipsychotic drugs and tricyclic antidepressants could be administered to fight with various forms of neuropathic pain. Posttraumatic sympathetic dystrophy, postmastectomy pain, postherpetic neuralgia, some forms of cancer pain, and other variants of neuropathic pain syndromes maybe addressed with novel adjuvant treatment protocols, planned on our cell-based TRPV1 assays, which may help to select among a number of empirically employed drugs (30+) and rationalize adjuvants' selection. Additional lead optimization may be enhanced with *in silico* screening of drugs available now (Figs. 10 and 11) or new drugs can reach the clinic sooner. For better specificity, however, we need to redirect drugs from the pleiotropic calmodulin target to docking site at the pore loop of TRPV1 (Figs. 10 and 11).

According to our results obtained in our TRPV1-NIH3T3/HaCaT and DRG cell-based functional assays, as well as, in laminectomised rat models, among clinically used pain adjuvant medications, either trifluoperazine (i.e. Terfluzine®) or chlorpromazine (i.e. Thorazine®) would be a better choice than amitriptyline. Amitriptyline and fluphenazine showed almost an order of magnitude less blocking efficacy among the registered drugs on TRPV1. Terfluzine® would be an adjuvant of choice in therapeutic protocols to potentiate either efficacy of opioids or NSAIDs, although, some other factors, for example inadvertent side effects, specificity to calmodulin and ADMETox characteristics can modify optimal selection. Calmidazolium, a miconazole type calmodulin antagonist was ranked to the 1^st^ place. We showed in laminectomised rat models that calmidazolium inhibits pain response to noxious heat, moreover firing evoked by NMDA and kainic acid. In the spinal dorsal horn calmidazolium inhibits other channels than the heat and inflammatory pain channel and it is active on NMDA and kainic acid receptors that usually prolong the duration of pain state. Calmidazolium, a not yet registered drug candidate, has previously been administered intrathecally in rat pain models and noted indeed analgesic [32]. Although was effective on the intrathecal route, some inadvertent action on locomotion has also been revealed. For human use, calmidazolium needs additional structural modifications, both with computational drug discovery and medical chemistry means [40].

Calmodulin kinase II (CaMKII) mediated intracellular signaling has recently been recognized to contribute to inflammation, pain and hyperalgesia [41] and activate TRPV1 in primary afferent neurons at the levels of spinal cord [11]. Although chemically different, calmidazolium and W-7, equally potent antagonists of calmodulin inhibit both phases of the formalin-induced abdominal pain and heat-induced tail-flick latency [32]. These *in vivo* observations also contradict to the Ca^2+^- calmodulin feedback theory of action of these agents. Animal studies, adjuvant therapy in the clinical practice and studies in this paper suggest that antipsychotic calmodulin antagonist drugs rather elicit *analgesia* instead of hyperalgesia and block Ca^2+^-entry in peripheral nociceptive neurons. calmodulin inhibitors showed non-competitive inhibitor kinetics with increasing dose of capsaicin. Our screening technology employs vanilloid/capsaicin-induced Ca^2+^-uptake assays in TRPV1 expressing cell lines, in the first round, then hits are further tested and validated in primary neuron cultures from rat embryonic DRG, enriched in TRPV1^+^ nociceptors.

A number of the Ca^2+^-transport experiments were carried out in “nominally zero Ca^2+^” buffer (2×10^−5^ M extracellular Ca^2+^ ([Ca^2+^]_o_) that reveals agonist-induced Ca^2+^-curents originating from either outside sources or the ER, then further amplified by Ca^2+^ induced Ca^2+^ release mechanism, has recently noted to occur in TRPV1 immunopositive nociceptor neurons. By Ca^2+^ induced Ca^2+^ release both TRPV1_PM_ and TRPV1_ER_ pools are actively involved in pain signaling [42], [43]. Ca^2+^ induced Ca^2+^ release is usually followed by a currently recognized mechanism, so called store operated Ca^2+^ entry, which replenish the ER store emptied by different Ca^2+^-channels, including the Ca^2+^/vanilloid-induced TRPV1_ER_. The usual function of store operated Ca^2+^ entry is to maintain Ca^2+^-homeostasis, around 1 mM in the ER, and [Ca^2+^]_i_ = 10^−7^ M, both essential for pain signaling in primary afferents sensory neurons. Coupling between Ca^2+^ induced Ca^2+^ release and store operated Ca^2+^ entry is now a well accepted hypothesis, therefore, [Ca^2+^]_o_ is an ideal test-bead for searching specific inhibitors affecting either one or both the TRPV1_PM_ and TRPV1_ER_ pools. Contrary to that noted with other TRP channels, calmodulin antagonists do not eliminate Ca^2+^-feedback. Even more, an initial observation with the camstatin peptide prompted us to hypothesise that antipsychotic inhibitors of calmodulin eliminate vanilloid-induced Ca^2+^-uptake due to binding to an extracellular domain of TRPV1. Likewise, calmidazolium may block noxious heat-, NMDA- and kainic acid -induced pain signaling by a mechanism that does not need internalization of the compound in laminectomised rat. Of importance, calmidazolium has explicit positive charge, and electrostatic interactions may be required for specific docking at the pore loop of TRPV1 (Figs. 10 and 11). Classical antipsychotic drugs, calmidazolium and camstatin may interact with TRPV1 on folds common in these Ca^2+^-binding proteins (i.e., at the Ca^2+^-filter/binding region).

By comparative homology search we identified a highly acidic domain near to the orifice of the Na^+^/Ca^2+^ channel. The^ 646^ ***D***L***E***FT***E***NY***D*** acidic tetrad of TRPV1 is unique and more extended stretch than that occurs in other TRPs in the TRPV1 (Fig. 10a). This motif is similar to the EF-hand (***D*** *X* ***D*** *X* ***D*** *GXX* ***D*** *XX* ***E***) and “*Excalibur*” (***D***X***D***X***D***XXXX***E***), both of them Ca^2+^ binding consensus domains, identified previously in calmodulin and bacterial calmodulin-like proteins, respectively [31]. Indeed, this acidic tetrad is targeted by other positively charged inhibitors such as the R_4_W_2_ peptide and ruthenium red. Both are well characterized inhibitors of capsaicin-induced Ca^2+^-current *via* TRPV1 channel. Our theory is that a short segment of the pore loop may mimic some feature of Ca^2+^-binding proteins where anti-calmodulin drugs dock and inhibit down-stream signaling (Fig. 10). Ruthenium red is widely used as a channel blocker in electrophysiology of sensory neurons, but only just recently has been mapped to bind at the acidic tetrad of TRPV1 (Fig. 10). Camstatin, a highly basic and lysine rich polypeptide may recognize this calmodulin-like region and block the TRPV1 channel without internalization. This particular domain of the pore loop may also serve as a cation filter and anti-calmodulin compounds can bind after Ca^2+^ binds first to this TRPV1 structure (Fig. 10), thereby preventing pore opening [44], [45]. Moreover, point mutatation studies established docking both ruthenium red and R_4_W_2_ to this structure. Conceivably, the most efficacious TRPV1 blockers have basic chemical character due to nitrogen heteroatoms in the structure that fit to the exposed acidic tetrad (Figs. 10 and 11) [45]. Experiments with the above mentioned mutants of Garcia-Martinez et al. and replacement experiment with radiolabeled R4W2 will provide the ultimate evidence for our claim based on the current experimental results and in silico docking studies.

Calmodulin, due to interaction with Ca^2+^, undergoes a robust conformational change and exposes hidden hydrophobic docking site for trifluoperazine and other conventional calmodulin antagonists [18], [19], [46]. By analogy, calmodulin-like mouth of the TRPV1 pore may react just like that described to other EF-hand structures [31] such as the S-100 Ca^2+^-binding proteins and “*Excalibur*”. Likewise, camstatin, an R/K-rich polypeptide may inhibit TRPV1 with a mechanism similar to other calmodulin antagonists of basic nature. The bulkiness of camstatin in fact has prompted us to hypothesize extracellular targeting of TRPV1 by anti-calmodulin drugs. Moreover, recently identified pore blockers from venom of a spider has further supported our TRPV1 channel blocker theory [46].

From selectivity point of view, calmidazolium, trifluoperazine, and W-7 have been determined inactive on intermediate Ca^2+^-activated K^+^ channels (IK_Ca_) of human erythrocytes, which indicates that these compounds can distinguish among different cation channels [47]. Although structurally distinct, both calmidazolium and W-7 are considered selective and potent inhibitors of calmodulin, yet they act extremely differently on the TRPV1 target, which is an important issue in future drug discovery. Both calmidazolium and W-7 were noted analgesic intrathecally in both phases of formalin tests in the rat [32]. Oral bioavailability of calmidazolium and additional derivatives, however, has yet to be evaluated in various pain models. Antipsychotic drugs selected on empirical bases can be ranked for rational adjuvants selection in various pain indications. Our data suggest that application of neuroleptics in severe cancer pain may enhance and complement efficacy of conventional painkillers by direct inhibition of TRPV1 Ca^2+^-channel, an alternative target not used by opiates and NSAIDs. Our data in this paper consistent with specific action of calmidazolium and other anti-calmodulin analogues on a short acidic domain of TRPV1, at the cation filter region not found in other TRP channels and Ca^2+^-binding polypeptides mimicking this region [46]. Better characterization of the extracellular calmodulin-like structure by molecular modeling and *in silico* screening might be exploited to design new generations of painkillers, thereby the novel but irreversible inflammatory pain neuron ablation technology [42], [48]–[50] can be supplemented with reversible channel blocker drugs both acting *via* TRPV1.

## Materials and Methods

### Agents and abbreviations:

CGP-37157, 7-Chloro - 5 - (2 - chlorophenyl) - 1,5 - dihydro - 4,1 – benzothiazepin - 2(3H) - one; KA, Kainate, [2S-[2α,3β,4β(1Z,3E,5R)]]-2-Carboxy-4-(5-carboxy-1-methyl-1,3-hexadienyl)-3-pyrrolidineacetic acid; M1, Miconazole 1; M2, Miconazole 2; M3, Miconazole 3; M4, Clotrimazole 4, M5, Clotrimazole 5; M6, Clotrimazole 6; M7, N-Benzyl Imidazole; SB 290157, N2-[(2,2-Diphenylethoxy)acetyl]-L-arginine; W-7, N- 6 – aminohexyl – 5 – chloro - 1 naphtalenesulfonamide; W-13, N - (4 - Aminobutyl) – 5 - chloro -2- naphthalenesulfonamide.

### Cell lines expressing TRPV1 ectopically

TRPV1-NIH3T3 mouse fibroblast line, ectopically expressing the rat TRPV1 was established previously. For validation of some data in human cells, a permanent TRPV1-HaCaT keratinocyte line was prepared by drug selection, similar to that described previously [1]–[4].

### Primary cultures expressing TRPV1 endogenously

Primary DRG cultures were prepared from E16 embryonic rats as described previously then plated in 15 cm Petri dishes for 4 h to remove the most adherent fibroblasts. For Ca^2+^-uptake studies primary afferents neurons cultured in 96 well plates (3×10^3^ cells/well), coated with poly-D lysine/collagen, which yielded both axons and dendrites bearing neurons due to neuronal growth factor supplementation in the culture medium [1]–[4]. The dendrites are large processes branching from the cell body that taper in diameter further from the cell body. The axons are a constant diameter and project over long distances from the cell body. Before the ion transport experiment, culture medium was removed and cells were washed three times. ^45^Ca-uptake assays were carried out in “nominally zero Ca^2+^” (i.e. 2×10^−5^ M extracellular Ca^2+^ ([Ca^2+^]_o_) in 25 mM Tris-HCl buffered Hanks' balanced salt solution (pH 7.5), supplemented with 10 µM CaCl_2_ and 0.8 mM MgCl_2_medium (HCM) [1]–[4].

### Vanilloid-induced ^45^Ca-uptake

Vanilloid agonist inducible calcium transport was assayed in adherent cell lines ectopically expressing the C-terminally ε-tagged rat TRPV1 (3×10^4^ cells/well) and primary cultures prepared from embryonic DRG, seeded in poly-D lysine/collagen coated 96 well plates. The ED_100_ for capsaicin-induction was determined to be ∼1 µM in TRPV1-NIH3T3 cells at pH 7.5. Therefore antagonist-screening assays were carried out with 2 µM capsaicin, an excess amount of agonist, which fully activates TRPV1 either in DRG neurons or in permanent cell lines ectopically expressing the vanilloid receptor, but does not cause Ca^2+^-cytotoxicity during the 10 min incubation period. To probe the TRPV1 specificity of capsaicin-induced ^45^Ca^2+^-uptake, experiments were carried out in the parental NIH 3T3 and HACAT cell lines and yielded negative results (data not shown). The previously established cell lines and standardized DRG cultures were extensively characterized for functional cell-based TRPV1 studies [1]–[4]. Immediately before the transport assay, cells were adapted to room temperature (20°C) for 5 min in HCM. ^45^Ca^2+^-uptake was performed for 10 min at 20°C in HCM using 0.1 µCi ^45^Ca^2+^ as radioactive tracer in a 100 µl final volume. To stop ^45^Ca^2+^-uptake, cells were rapidly changed back into 0.2 ml HCM, washed 4 additional times with 0.2 ml HCM, and then lysed in 80 µl/well RIPA buffer (50 mM Tris-HCl, pH 7.5, 150 mM NaCl, 1% Triton X-100, 0.5% deoxycholate, 0.1% SDS, 5 mM EDTA) for 30 min [1]–[4]. Aliquots of the solubilized cell extracts were mixed with 120 µl Microscint-40 and counted in a 96 well plate liquid scintillation counter (TopCount-NXT, Packard).

### Anesthesia and surgery

Extracellular single-neuron recordings were made in chloral hydrate-anesthetized (4 g/kg initial dose, *i.p.*, with supplemental doses as required) male Wistar rats weighing between 350–450 g. The head of the animal was mounted in a stereotaxic frame. The lumbar enlargement (L3–L5 segments) was exposed by a laminectomy and the spinal cord was covered with a pool of warmed mineral oil to prevent drying. Rectal temperature as well as the temperature of the mineral oil were monitored with temperature probes and kept at 37°C by a warm water-heated blanket beneath the rat and an infrared heat lamp from above. The level of anesthesia was maintained so that the rats showed no sign of discomfort but the tail flick reflex could be evoked by application of noxious heat (43–45°C) or mechanical stimulus (squeeze) to the tail. Recordings were only commenced at least 1 h after surgery. All animal experiments were performed in accordance with institutional animal welfare guidelines.

### Extracellular recordings and iontophoresis

Compound recording/iontophoresis electrodes were made of a seven barreled array of thin wall borosilicate glass capillary tubings (Kation Scientific, Minneapolis, MN) The recording barrel contained a low impedance (1 MΩ) 7-µm carbon fiber and drugs were delivered from the surrounding barrels. Single unit extracellular recordings were made from selected dorsal horn neurons characterized by their responses to both innocuous (brush, pressure) and noxious (pinch, squeeze that was felt as painful by the experimenter) stimuli applied to the excitatory receptive fields of the hind paw. Mechanical stimuli of increasing strength were delivered by a small brush and three serrated clamps with a graduated force. The experimenter released clamps so that force was standard for each mechanical stimulus delivered over a period of 3 s, and about 1 min were allowed to pass between trials. Neurons were characterized as low threshold (LT), nociceptive specific (NS) or wide dynamic range (WDR) by their responses to mechanical stimuli of increasing strength. Extracellular signals from dorsal horn neurons were amplified and filtered (300 Hz to 8 KHz) using an ExAmp-20K equipment and preamplifier (Kation Scientific). Neuronal activity was displayed on an oscilloscope and through an audio analyzer and detected with a WD-2 window discriminator (Dagan, Minneapolis, MN). The number of action potentials per second was counted by the computer and the resulting peristimulus time histograms were displayed. Iontophoretic drug delivery and collection of experimental data were performed by a multifunction data acquisition board (PCI-1200, National Instruments, Austin, TX) placed in a computer and programmed in LabVIEW 7 (National Instruments). Delivery of drugs by microiontophoresis was performed using Union-36 constant current source units (Kation Scientific). Drug barrels of the combined electrodes contained one of the following freshly made solutions: 100 mM N-methyl-D-aspartate Na (NMDA) in 100 mM NaCl (pH 8.0), 20 mM kainic acid in 100 mM NaCl (pH 8.0), 5 mM calmidazolium chloride in 100 mM NaCl (pH 7.2) was prepared using a 100 mM calmidazolium in ethanol stock solution. NMDA and kainic acid were ejected with negative iontophoretic currents ranging from 10 to 100 nA for 5 s in every 2 min. calmidazolium was ejected with positive currents at 100 nA for 120 s. Retaining currents of opposite directions between 3–10 nA were used for all drugs.

### Heat stimulation

Single unit extracellular recordings were made from selected dorsal horn neurons responding to noxious heat delivered by a projector lamp focused on the blackened surface of the hind paw. Skin temperature was monitored using an infrared thermometer. Temperature ramps were generated from a holding temperature of 30°C to a peak of 50°C at a rate of about 2°C/s. The heat stimulus was turned off when skin temperature reached 50°C.

### Data analysis

Statistical evaluations were made using the total number of spikes minus background activity evoked during each epoch of excitation by heat stimuli or iontophoretic application of an excitatory compound. The background neuronal discharge was calculated by averaging a 15 s period of ongoing activity preceding each epoch of excitation and this value was subtracted from the total number of evoked spikes. Differences in magnitude between different response epochs of a single cell were confirmed by one-factor analysis of variance (with Student-Newman-Keuls test for post-hoc analysis) by comparing the total number of spikes per excitation period. To make data from different experiments more comparable, analysis of pooled data was done after normalizing the baseline stimulus-evoked response to 100%. Means±SD of a number (*n*) of observations are given throughout. A *P* value of <0.05 was considered significant in all cases.

### Molecular modeling of TM5-pore loop-TM6 domain of TRPV1 and docking calmidazolium and chlorpromazine to the extracellular portion

The preliminary channel domain model was used in a fixed protein flexible ligand docking protocol using simulated annealing as it is implemented in the MOE 2004.03 program package from Chemical Computing Group. The homotetramer of the TM5-pore loop-TM6 domain of TRPV1 was built using the Swiss-PdbViewer molecular graphics and the SWISS-MODEL comparative homology modeling program package [51]–[53]. The Q8NER1 sequence from TrEMBL [54] was used as a target protein. Transmembrane prediction using the TMHMM 2.0 server [55] was carried out to delineate the transmembrane segments. The predicted extracellular pore loop together with the fifth and sixth transmembrane helices were extracted from the full length protein sequence and aligned with the sequence of the bacterial K^+^ channel KcsA [56]. The X-ray crystal structure of the M1-pore loop-M2 of the KcsA was used (PDB accession code 1K4C) as a template for homology modeling. Optimization of the loop regions was carried out by applying extensive constraint minimization using the GROMOS96 [57] force-field implemented in Swiss-PdbViewer. The homotetramer structures were built manually using the non-crystallographic symmetry operators derived from the template. The final depictions of the S5-pore loop-S6 segment of TRPV1 model were produced using the VMD program [58]. The possible interactions between the channel region of the tetrameric TRPV1 receptor complex and the inhibitors were calculated with the Sybyl molecular modeling package (litSYBYL molecular modeling software, version 7.1, Tripos Associates Inc., 1699 Hanley Rd, St. Louis, MO 63144-2913.) using the general Tripos force field for the ligands containing chemical bonds unusual in biological macromolecular modeling. The structure of the receptor-ligand complex was approximated by minimizing with the prepositioned ligand. The backbone of the residues of the receptor complex outside the 20 angstrom boundary from the inhibitor molecule were constrained in a fixed position allowing larger movement of the neighboring amino acids only. The calmidazolium, holding an inherent positive charge, was fixed by closely surrounding Asp and Glu residues. No aromatic-aromatic interactions were observed indicating the dominance of charged residues in the channel blocking process.
